# Intravenous thrombolysis versus dual antiplatelet therapy for patients with acute minor ischaemic stroke: a systematic review and meta-analysis

**DOI:** 10.3389/fphar.2024.1377475

**Published:** 2024-06-10

**Authors:** Bin Qin, Lin Fu, Huixun Qin, Yuming Liang, Cheng Qin, Jiede Zhang, Wen Gao

**Affiliations:** ^1^ Department of Neurology, Liuzhou People’s Hospital, Liuzhou, Guangxi, China; ^2^ The First Affiliated Hospital of Guangxi Medical University, Nanning, Guangxi, China; ^3^ Liuzhou Key Laboratory of Epilepsy Prevention and Research, Liuzhou, Guangxi, China

**Keywords:** intravenous thrombolysis, dual antiplatelet therapy, minor stroke, acute ischaemic stroke, meta-analysis

## Abstract

**Background and purpose:**

The efficacy of intravenous thrombolysis (IVT) in patients with acute minor ischaemic stroke (AMIS) remains unclear. We performed a meta-analysis to compare the efficacy and safety of IVT and dual antiplatelet therapy (DAPT) in patients with AMIS.

**Methods:**

The Embase, Cochrane Library, PubMed, and Web of Science databases were searched up to 10 October, 2023. Prospective and retrospective studies comparing the clinical outcomes of IVT and DAPT were included. Odds ratios (ORs) and 95% confidence intervals (CIs) for early neurological deterioration (END), excellent and favourable functional outcomes, recurrent ischaemic stroke at 3 months, mortality at 3 months, and symptomatic intracranial haemorrhage (ICH) were pooled using a random-effects model.

**Results:**

Of the five included studies, 6,340 patients were included. In patients with AMIS, IVT was not significantly associated with excellent and favourable functional outcomes, recurrent ischaemic stroke, or all-cause mortality at 3 months compared to early DAPT. However, a higher risk of symptomatic ICH (OR, 9.31; 95% CI, 3.39–25.57) and END (OR, 2.75; 95% CI, 1.76–4.30) were observed with IVT.

**Conclusion:**

This meta-analysis indicated that IVT was not superior to DAPT in patients with AMIS, especially in those with nondisabling AIS. However, these findings should be interpreted with caution and have some limitations. Further, well-designed randomised controlled trials are warranted.

## Introduction

An estimated 50% of stroke patients have mild neurologic deficits [National Institutes of Health Stroke Scale (NIHSS) score ≤5] on admission (Saber and Saver, 2020; Xiong et al., 2021). However, stroke with a low NIHSS score is not necessarily nondisabling; approximately one-third of them cannot achieve functional independence (Nedeltchev et al., 2007; Smith et al., 2011; Ali et al., 2016). Acute minor ischaemic stroke (AMIS) remains at high risk of poor function and recurrence in the short- and long-term (Wang et al., 2015a; Wang et al., 2015b). Identifying secondary prevention strategies in this population is crucial to reducing morbidity and mortality. Current guidelines recommend intravenous (IV) alteplase for patients with acute ischaemic stroke (AIS) within 4.5 h of the onset of symptoms (Powers et al., 2019; Liu et al., 2020; Berge et al., 2021). Many major alteplase trials excluded AIS with low NIHSS scores, resulting in a poor understanding of the safety and efficacy of intravenous thrombolysis (IVT) (Lees et al., 2016). Several retrospective studies found that IV alteplase was effective for AMIS (Köhrmann et al., 2009; Hassan et al., 2010; Choi et al., 2015; Laurencin et al., 2015), while others did not (Rajajee et al., 2006; Khatri et al., 2010; Huisa et al., 2012; Frank et al., 2013). In addition, meta-analyses also yield conflicting results (Shi et al., 2014; Yeo et al., 2014; You et al., 2018; Lan et al., 2019). A recent randomised controlled trial (RCT) was to test the efficacy and safety of alteplase versus aspirin alone in patients with AMIS and 3 h after the onset of ischaemic stroke symptoms. The results showed that no significant differences were found between the two groups for 90-day functional outcomes, but alteplase caused more symptomatic intracerebral haemorrhage. Despite this, the conclusion of this trial should be drawn with caution due to its premature termination. Therefore, the evidence in support of IVT for AMIS remains inconclusive.

Dual antiplatelet therapy (DAPT) is another acute treatment option for patients with AMIS. The Clopidogrel with Aspirin in Acute Minor Stroke or Transient Ischemic Attack (CHANCE) trial indicated that the combination of clopidogrel and aspirin reduces the risk of stroke in the first 90 days and does not increase the risk of haemorrhage in patients with AMIS within 24 h of symptom onset compared to aspirin alone (Wang et al., 2013). The Clopidogrel and Aspirin in Acute Ischemic Stroke and High-Risk TIA (POINT) trial also confirmed that the combination of clopidogrel and aspirin had a lower risk of major ischaemic events in patients with AMIS (Johnston et al., 2018). Furthermore, CHANCE showed that DAPT reduces recurrent stroke most effectively within the first 2 weeks of treatment (Pan et al., 2017). Compared to antiplatelet monotherapy, early DAPT prevents further vascular events and improves functional outcomes after AMIS.

In this context, the question is: For patients with AMIS, what is the best treatment strategy: IVT or early DAPT? Currently, there is no definitive evidence to compare IVT with early DAPT in patients with AMIS. To aid decision-making in clinical practice, we performed a systematic review and meta-analysis of all published studies that compared the efficacy and safety of IVT with early DAPT in patients with AMIS.

## Materials and methods

This meta-analysis was carried out according to the Preferred Reporting Items for Systematic Reviews and Meta-Analyses guidelines (PRISMA) (Page et al., 2021).

### Search strategy and eligibility criteria

Electronic searches of Embase, Cochrane Library, PubMed, and Web of Science were performed to identify all relevant studies. The last search was carried out in 10 October, 2023 using the following terms: (thrombolysis* OR “intravenous tissue plasminogen activator” OR rt-PA OR t-PA OR alteplase* OR tPA OR tenecteplase OR TNK OR TNKase) AND (Antiplatelet OR Anti-platelet OR aspirin OR acetylsalicylic acid OR ASA OR Clopidogrel OR Plavix OR Iscover OR thienopyridines OR ADP receptor inhibitors OR Ticagrelor OR Brilique OR Brilinta) AND (“rapidly improving symptoms” OR “nondisabling deficit” OR mild OR minor OR minimal OR “low NIHSS” OR “low National Institutes of Health Stroke Scale” OR “NIHSS ≤5” OR “NIHSS <6” OR “NIHSS ≤3” OR “NIHSS 0-5” OR “NIHSS 0-3”) AND (stroke OR cerebral ischemia) (Supplementary Table S1). No language restrictions were imposed. Furthermore, the reference lists of the included studies and those of previous reviews, editorials, and meta-analyses were manually reviewed.

### Study selection, data extraction and quality assessment, and study outcomes

Studies that met the following criteria were included: 1) studies with prospective and retrospective, randomised and nonrandomised designs; 2) patients with AIS and NIHSS scores ≤5; 3) studies in which patients were assigned to two groups, one group receiving IVT and the other group receiving early DAPT; and 4) studies reporting at least one functional outcome at 3 months. The following exclusion criteria were used: 1) studies with <10 participants in each arm, 2) insufficient data, and 3) reviews, letters, conference abstracts, and case reports. Two experienced investigators (BQ and LF) independently screened the abstracts and titles of the search results and determined the eligibility of candidate studies. Any discrepancies were resolved by discussion with other investigators (WG and HQ).

Two investigators (BQ and LF) independently extracted summary data for analysis. A standardised form was used to extract the following data: identity of the first author, publication date, type of study, location of the study population, age of the study population, female ratio, NIHSS score at baseline, vessel occlusion sites, functional outcome at 3 months, early neurological deterioration (END), symptomatic intracranial haemorrhage (ICH), mortality at 3 months, and other related detailed characteristics of the included studies. The quality assessment of the included studies was presented in a way consistent with the Cochrane Collaboration’s tool to assess the risk of bias, version 2.0 (RoB 2.0) for RCTs and Risk Of Bias In Nonrandomized Studies of Interventions (ROBINSI) for nonrandomized studies. Each eligible study was independently examined by two investigators (BQ and LF) and disagreements were resolved by discussion with a third investigator (WG).

The primary outcome was an excellent functional outcome [modified Rankin scale (mRS) score of 0–1 at 3 months]. Secondary outcomes were favourable functional outcomes (mRS scale scores of 0–2) at 3 months, END at 24 h (defined as an increase of ≥2 points in the NIHSS score, but not due to cerebral haemorrhage), recurrent ischaemic stroke at 3 months, and all-cause mortality at 3 months.

### Data analysis

Pooled odds ratios (ORs) with 95% confidence intervals (CIs) were calculated for each outcome in patients receiving IVT and those receiving early DAPT using random-effects models. For primary efficacy and safety outcomes (excellent functional outcomes and symptomatic ICH), we also calculated the pooled adjusted OR (multiple regression or matching analyses) when reported. We used the logarithmic-adjusted OR and the corresponding standard errors to calculate the pooled adjusted OR in a random-effects analysis. Logarithmic ORs were calculated with lnOR; standard errors were calculated using (upper CI–lower CI)/3.92. Heterogeneity between studies was evaluated using the *p*-value of the χ^2^ statistics. I^2^ statistics were used to quantify heterogeneity between studies. Mild, moderate, and high heterogeneity was identified, with I^2^ values of approximately 25%, 50%, and 75%, respectively (Higgins JPT et al., 2019). In order to account for heterogeneity, subgroup analyses were performed by NIHSS scores at admission (NIHSS ≤ 3 or NIHSS ≤ 5) because the cut-off NIHSS score of 0–3 was chosen according to RCTs that introduced the use of DAPT in the acute phase of mild stroke (Wang et al., 2013; Johnston et al., 2018), and onset to treatment time into DAPT group (≤4.5 h or ≤ 24 h). Sensitivity analysis by excluding studies only including patients with large vessel occlusion (LVO) was performed to assess the robustness of the results. All statistical tests were two-sided, with a significance threshold of *p* < 0.05. A funnel plot of the reported effect estimates was used to assess the risk of publication bias and the Egger’s regression test was used to evaluate the presence of publication bias. All data were analysed using Review Manager (RevMan) (version 5.4; the Cochrane Collaboration, 2020).

## Results

### Study selection and characteristics

**FIGURE 1:**
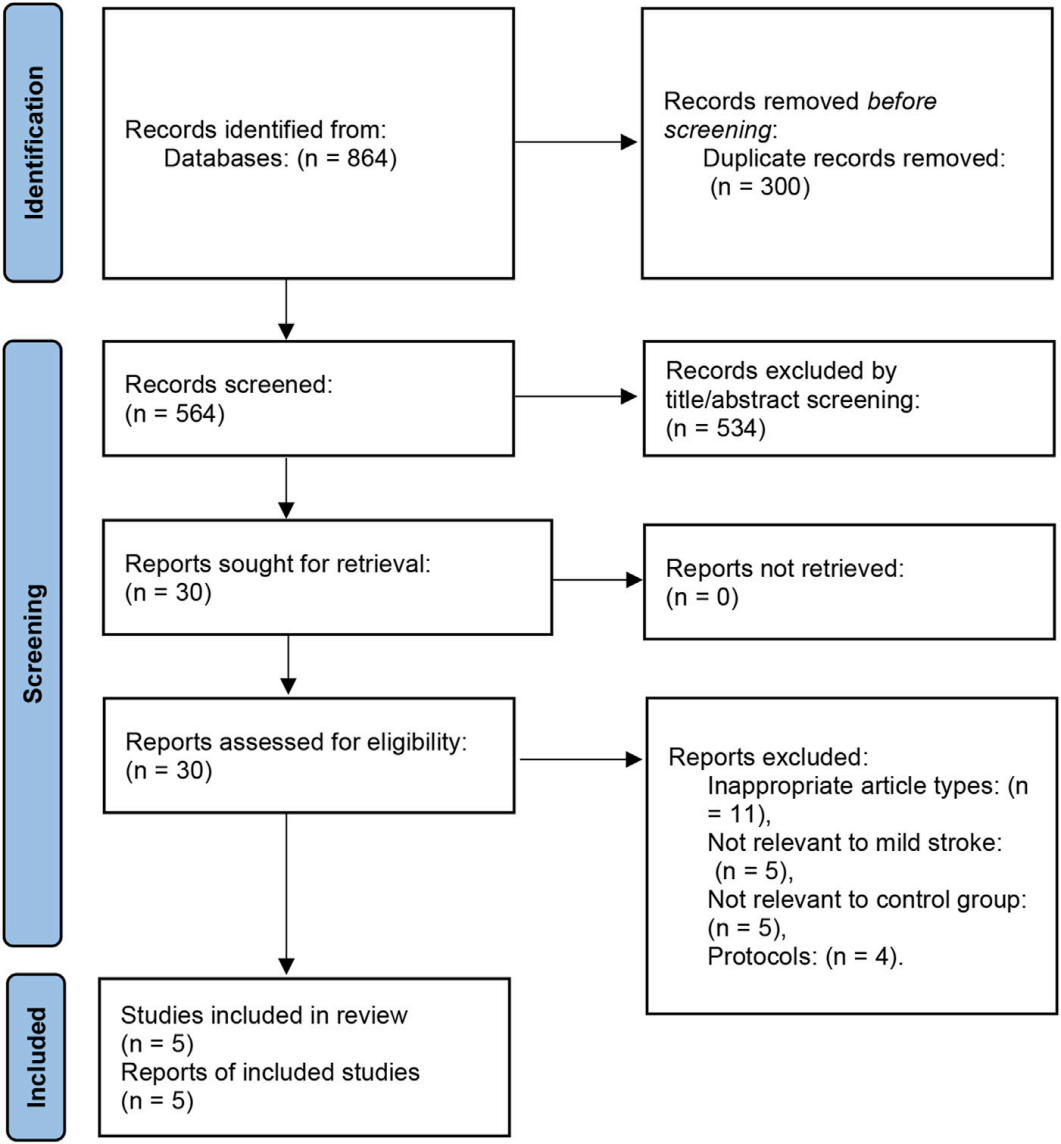
Flow chart of the selection process in this meta-analysis.

A total of 864 records were searched using electronic databases, of which 300 were excluded because they were duplicates. After retrieving 30 articles for full-text review, 25 articles were excluded for several reasons: inappropriate article types [reviews, systematic reviews, and meta-analyses] (*n* = 11), patients with non-AMIS (*n* = 5), control group that was not eligible (*n* = 5), and protocols (*n* = 4). Finally, five articles (one RCT, two prospective studies, and two retrospective studies) were included after qualitative and quantitative analyses, containing 6,340 patients (Lan et al., 2020; Wang et al., 2021; Chen et al., 2023; Duan et al., 2023; Sykora et al., 2023). The PRISMA flow chart of the study inclusion process can be seen in Figure 1.

The main characteristics of the studies included in this meta-analysis are summarised in Table 1. A total of 2,290 patients in the IVT group and 4,050 patients in the DAPT group were included in the quantitative analysis. The median age ranged from 61.0 to 68.1 years in the IVT group and from 63.0 to 70.8 years in the DAPT group. Females represented 35.2% of the patients. All studies used alteplase as a thrombolytic agent and a combination of aspirin and clopidogrel as DAPT. The median baseline NIHSS score ranged from 1 to 4 points and was predominantly 2 points. Of the 5 studies, 2 included patients with nondisabling AIS (Chen et al., 2023; Duan et al., 2023), one included patient with disabling AIS (Duan et al., 2023), and others did not report the relevant information. Only one study just included patients with LVO (Duan et al., 2023).

**TABLE 1:** Details of studies included in the meta–analysis.

Author and year	Countries	Study design	MIS definition	Localization	IVT					DAPT				
					No. of patients	Age (year)^a^	Female (%)	NIHSS at admission^a^	OTT (hours)	No. of patients	Age (year)^a^	Female (%)	NIHSS at admission^a^	OTT (hours)
Chen et al. (2023)	China	RCT	NIHSS ≤ 5	AC + PC	350	64 (56–71)	31.4	2 (1–3)	≤4.5	369	65 (57–71)	30.6	2 (1–3)	≤4.5
Duan et al. (2023)	China	PO–NR	NIHSS ≤ 5	AC + PC	251	62.0 (55.0–68.0)	29.1	3.0 (2.0–4.0)	≤4.5	722	63.0 (56.0–70.0)	29.4	2.0 (1.0–4.0)	≤4.5
Lan et al. (2020)	China	RO–NR	NIHSS ≤ 5	NA	109	67 (33–89)	32.1	4 (1–5)	≤4.5	119	64 (32–86)	27.7	2.5 (0–5)	≤4.5 or ≤ 24
Sykora et al. (2023)	Austria	PO–NR	NIHSS ≤ 3	AC + PC	1,195	68.1 (21–98)	37.2	2 (0–3)	≤4.5	2,625	70.8 (19–99)	38.7	1 (0–3)	≤24
Wang et al. (2021)	China	RO–NR	NIHSS ≤ 3	NA	385	61.0 (54.0–69.0)	33.2	2 (1–3)	≤4.5	215	63.8 (55.2–71.2)	30.7	2 (1–3)	≤4.5 or ≤ 24

^a^ Median [interquartile range (IQR)] reported.

AC, anterior circulation; DAPT, dual antiplatelet therapy; IVT, intravenous thrombolysis; MIS, minor ischemic stroke; NA, not available; NIHSS, national institutes of health stroke scale; OTT, onset to treatment; PC, posterior circulation; RCT, randomized controlled trial; RO–NR, retrospective nonrandomized; PO–NR, prospective, nonrandomized.

### Risk of bias and publication bias

The risk of bias for each study using the RoB 2.0 or ROBINS-I tool and in all studies was an overall variable, as shown in Supplementary Table S2. Most studies had a low-to-moderate risk of bias. Visual inspection of funnel plots and calculation of Egger test results were not reported because no more than ten studies reported each main outcome.

### Primary efficacy outcome

**FIGURE 2:**
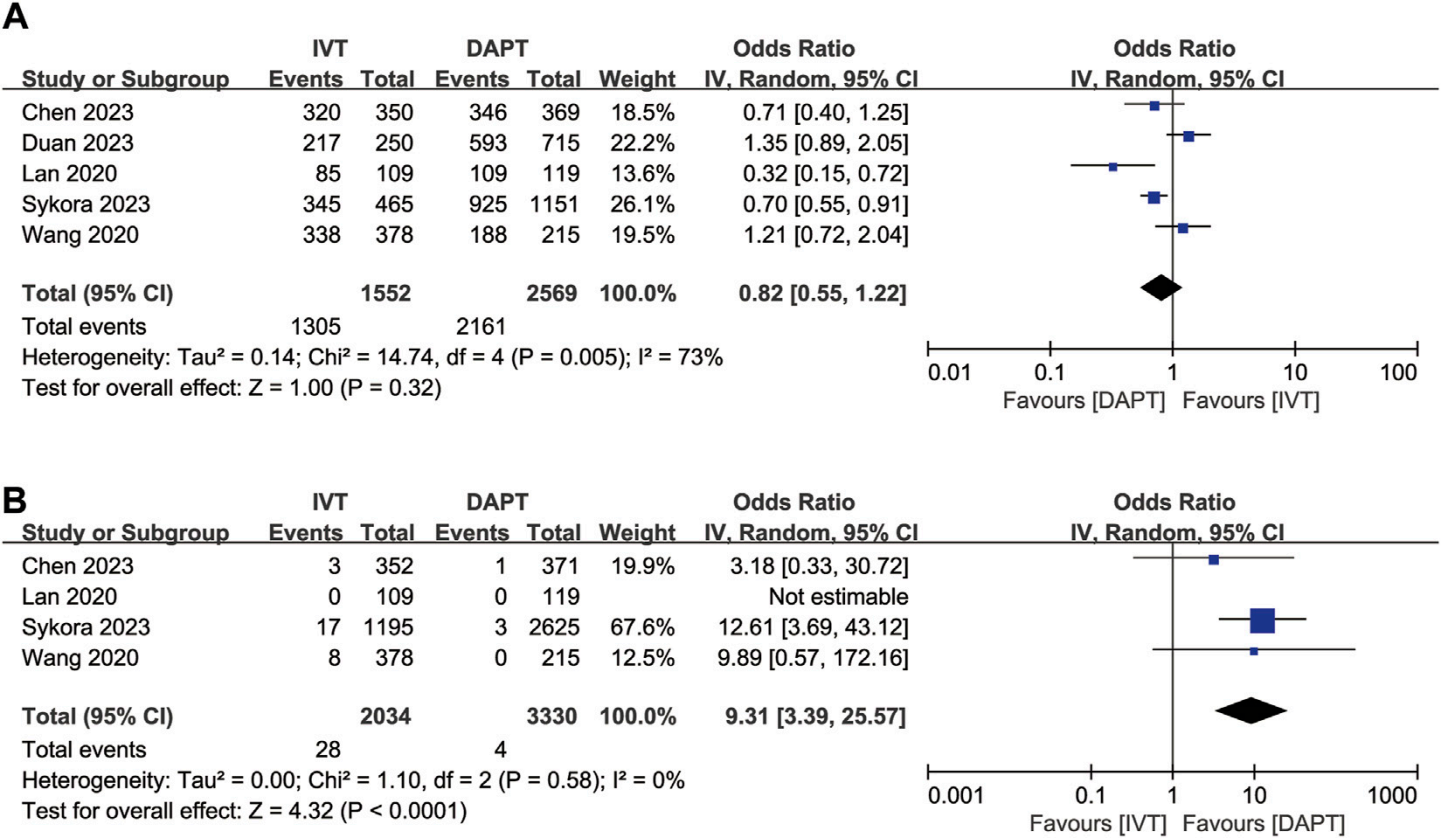
Forest plot of the odds ratios of primary efficacy and safety outcomes in patients with acute minor ischaemic stroke: **(A)** excellent functional outcome (mRS 0–1 at 3 months); **(B)** symptomatic ICH. CI, confidence interval; ICH, intracranial haemorrhage; IV, inverse variance; mRS, modified Rankin scale; SE, standard error.

Excellent functional outcome data (mRS score 0–1 at 3 months) were available for five of the included studies. IVT was not significantly associated with excellent functional outcome compared to early DAPT (OR, 0.82; 95% CI, 0.55–1.22; *p* = 0.32; Figure 2A). After adjustment, IVT was also not significantly associated with excellent functional outcome (OR, 0.83; 95% CI, 0.55–1.25; *p* = 0.38; Supplementary Figure S1A).

### Safety outcome

Symptomatic ICH was available for four of the included studies. IVT was associated with symptomatic ICH (OR, 9.31; 95% CI, 3.39–25.57; *p* < 0.0001; Figure 2B) compared to early DAPT. After adjustment, IVT was also associated with a higher risk of symptomatic ICH (OR, 6.74; 95% CI, 2.41–18.84; *p* = 0.0003, Supplementary Figure S1B).

### Secondary efficacy outcomes

**FIGURE 3:**
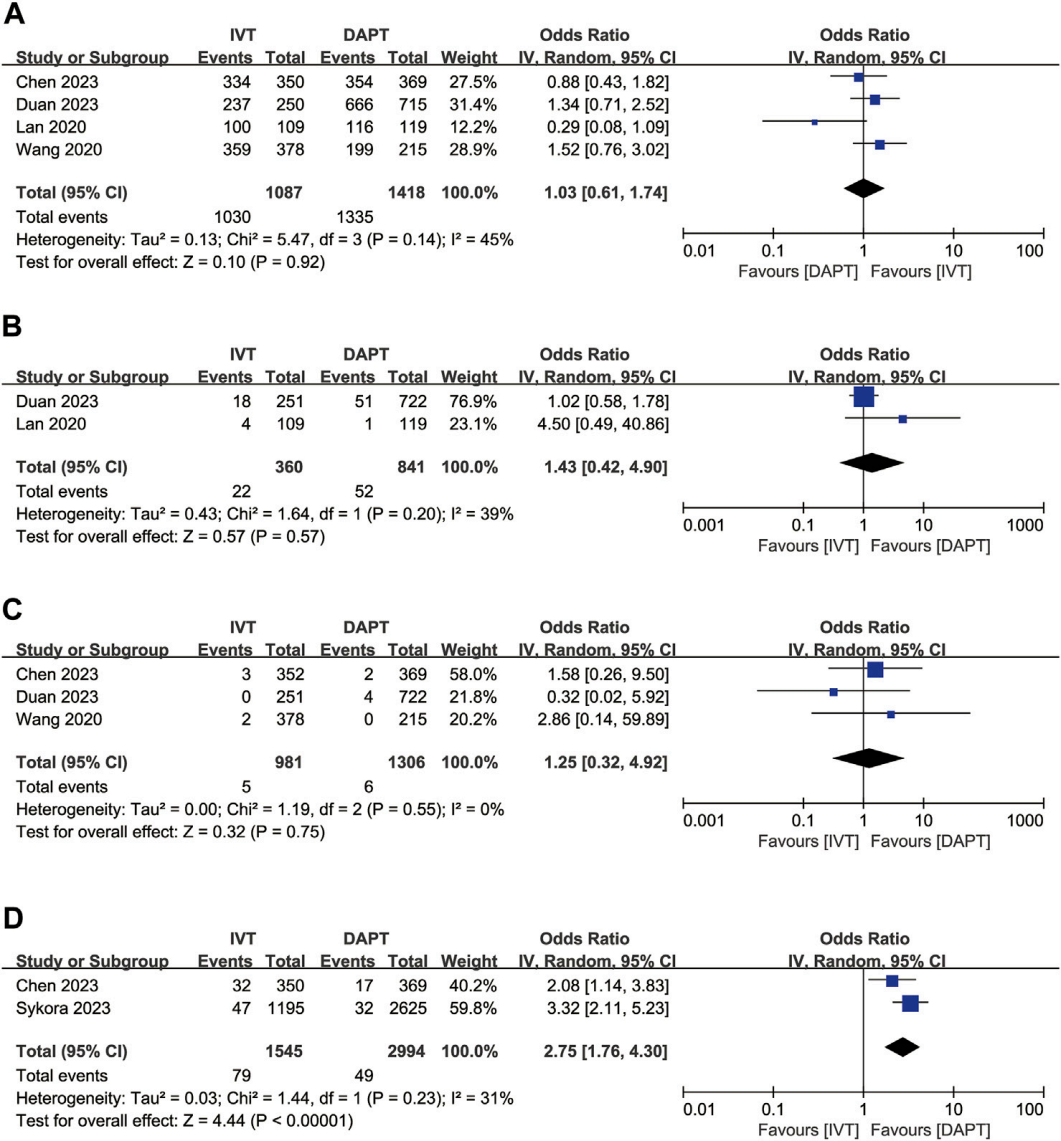
Forest plot of the odds ratios of the secondary efficacy outcomes in patients with acute minor ischaemic stroke: **(A)** favourable functional outcome (mRS 0–2 at 3 months); **(B)** recurrent ischaemic stroke at 3 months; **(C)** all-cause mortality at 3 months; **(D)** early neurologic deterioration. CI, confidence interval; IV, inverse variance; mRS, modified Rankin scale; SE, standard error.

For the favourable functional outcome (mRS score 0–2 at 3 months), IVT did not show significant differences compared to early DAPT (OR, 1.03; 95% CI, 0.61–1.74; *p* = 0.92; Figure 3A). IVT was also not significantly associated with recurrent ischaemic stroke at 3 months (OR, 1.43, 95% CI 0.42–4.90; *p* = 0.57; Figure 3B) and all-cause mortality at 3 months (OR, 1.25; 95% CI, 0.32–4.92; *p* = 0.75; Figure 3C). However, IVT was associated with a higher risk of END (OR, 2.75; 95% CI, 1.76–4.30; *p* < 0.00001; Figure 3D) compared to early DAPT.

### Sensitivity analysis and subgroup analysis

In the subgroup analyses, studies were categorized by NIHSS scores at admission and onset to treatment time into DAPT group. The results of the subgroup analyses are shown in Supplementary Figures S2, S3. In the subgroup analysis by NIHSS scores at admission, there was no significant difference in excellent functional outcome between patients with NIHSS scores of 0–3 and NIHSS scores of 0–5 at admission (Supplementary Figure S2). Moreover, no significant association was observed between excellent functional outcome and onset to treatment time into DAPT group (≤4.5 h: OR, 1.01; 95% CI, 0.54–1.89; *p* = 0.98 versus ≤ 24 h: OR, 0.70; 95% CI, 0.40–1.23; *p* = 0.21; Supplementary Figure S3). A sensitivity analysis by omitting one study only including patients with LVO was performed (Supplementary Figure S4), yielded similar results to the overall analyses for excellent functional outcome. The analysis results reflected that the results were statistically stable and reliable.

## Discussion

The present meta-analysis showed that IVT was not superior to early DAPT in terms of functional outcomes among patients with AMIS. A higher number of cases of END and symptomatic ICH were observed in the IVT group. There were no significant differences between the two groups in other secondary efficacy outcomes or subgroup analyses.

To the best of our knowledge, the PRISMS study was the first randomised multicentre trial to investigate the effects of IV alteplase versus single antiplatelet therapy in patients with AMIS (Khatri et al., 2018). However, the study was terminated early, rendering the results inconclusive. Based on these results, IVT is not recommended for patients with AMIS. However, a large series of nonrandomised suggested the benefit of IVT in patients with AMIS (Greisenegger et al., 2014; You et al., 2018; Sykora et al., 2023). Furthermore, based on a subgroup analysis of patients with AMIS in the IST-3 trial, IVT was superior to standard medical treatment (Khatri et al., 2015). IVT in patients with AMIS remains controversial. On the contrary, early DAPT has been shown to prevent further vascular events after mild stroke and is superior to monotherapy with antiplatelet agents (Wang et al., 2013; Johnston et al., 2018; Prasad et al., 2018; Amarenco et al., 2020). POINT confirmed that clopidogrel plus aspirin was associated with a lower risk of major ischaemic events in patients with AMIS or high-risk TIA who could be treated within 12 h after the onset of symptoms, but a higher risk of major haemorrhage at 90 days than aspirin alone in such patients (Johnston et al., 2018). Furthermore, the CHANCE study showed the superiority of clopidogrel plus aspirin in patients with TIA or AMIS who could be treated within 24 h of the onset of symptoms (Wang et al., 2013). Therefore, it is important to investigate which strategy (IVT or early DAPT) should be used to treat AMIS (Chen et al., 2023). Our results showed that among patients with AMIS, IVT was not superior to early DAPT in terms of primary outcome of excellent functional outcomes. Our results are in line with those of previous studies, suggesting non-superiority in patients with low NIHSS stroke undergoing thrombolytic therapy (Khatri et al., 2018; Sykora et al., 2022). Moreover, most patients in this meta-analysis were nondisabling AIS and mild neurological deficits. This is consistent with the results of the PRISMS trial, which showed that IV alteplase was not superior to aspirin in improving functional outcomes in nondisabling stroke (Khatri et al., 2018).

END appears to be of particular interest regarding secondary efficacy outcomes. Our study showed higher END in patients who underwent IVT than in those who underwent early DAPT. A previous study reported that DAPT can decrease the frequency of END (Berberich et al., 2019). Another recent study indicated that acute DAPT is associated with a reduced risk of END (Vynckier et al., 2021). Interestingly, in our study, early DAPT appeared to decrease the risk of END compared to IVT, although the rates of END are comparable to those previously described in patients with AMIS (13.3%) (Tang et al., 2021) and in unselected stroke patients (6.7%) (Yu et al., 2020) after intravenous alteplase. The lack of antithrombotic effects in 24 h, considering the short half-life of the drug, could contribute to stroke recurrence or progression of the thrombus (Chen et al., 2023). However, the definition of END varies in the included studies, which may have led to the heterogeneity and the results have to be interpreted with caution. Future research with consistent definition of END is required to better demonstrate our findings.

In terms of safety outcomes, compared to early DAPT, there was an increased risk of symptomatic ICH in the IVT group. The 0.12% rate of symptomatic ICH with early DAPT in this study was comparable to the frequency previously described (0.10%–0.20%) (Wang et al., 2013; Johnston et al., 2018). Similarly, the rate of symptomatic ICH with alteplase was 1.38% in this study, which was similar to other studies that included patients with minor stroke treated with alteplase (2.4%–3.7%) (Greisenegger et al., 2014; Shi et al., 2014; Tsivgoulis et al., 2020). However, our study indicated that IVT was associated with symptomatic ICH compared to early DAPT. END seems to be particularly relevant in this context. The slightly increased risk of symptomatic ICH may contribute to the development of END in patients undergoing IVT.

Our study had some limitations. First, although the present meta-analysis had a large sample size, it reported an adjusted effect size for confounding factors. However, most of the included studies were nonrandomised design. It is limited by potential unmeasured residual confounding factors, such as selective bias, that can influence the reported results, hence our findings should be interpreted with caution. However, evidence derived from nonrandomised design might allow greater similarities with real-world clinical practice. Second, some studies did not record the aetiology of stroke, which, in turn, could not be included in the analysis. This limits the ability to provide a detailed interpretation of the data. Third, in some study, the onset of treatment time was less than 4.5 h of the last known well in the alteplase group, but less than 24 h in the DAPT group, which may have led to a potential bias in the reported results. However, the subgroups of onset to treatment time into DAPT group (less than 4.5 h of symptom onset versus 24 h of symptom onset) did not differ significantly, which remained similar results to the overall analyses; thus, the results of this meta-analysis appeared to be stable and reliable. Fourth, the subgroup analysis of nondisabling versus disabling AIS was not performed due to only one study reported the outcomes in patients with disabling AIS. Nevertheless, most patients in this meta-analysis were nondisabling AIS and mild neurological deficits [NIHSS score ≤3] (Wang et al., 2021; Chen et al., 2023; Duan et al., 2023; Sykora et al., 2023). Thus, non-disabling stroke may be seen as representative in this meta-analysis. Finally, alteplase was the only thrombolytic agent used in the included studies. Therefore, tenecteplase may serve as an effective thrombolytic agent. In such cases, additional studies are necessary to determine whether tenecteplase is more effective than early DAPT in the treatment of AMIS.

## Conclusion

The present meta-analysis indicated that IVT does not appear to have better safety or efficacy compared to early DAPT in patients with AMIS, especially in those with nondisabling AIS. However, these findings should be interpreted with caution and have some limitations. More well-designed RCTs are warranted.

## Data Availability

The raw data supporting the conclusion of this article will be made available by the authors, without undue reservation.
