# Supplementary material for: Intravenous thrombolysis versus dual antiplatelet therapy for patients with acute minor ischaemic stroke: a systematic review and meta-analysis
Source: Front Pharmacol. 2024 Jun 10;15:1377475. doi: 10.3389/fphar.2024.1377475 (PMC11194400; doi:10.3389/fphar.2024.1377475)
Supplement: Supplementary file 1 [file DataSheet1.docx]

Supplementary Material

# Supplementary Tables and Figures

## Supplementary Tables

**Supplementary** **Table 1.** Search strategy in PubMed database

| **Search number** | **Search Details** |
| --- | --- |
| 1 | Stroke[Title/Abstract] OR cerebral ischemia[Title/Abstract] |
| 2 | rapidly improving symptoms[Title/Abstract] OR nondisabling deficit[Title/Abstract] OR mild[Title/Abstract] OR minor[Title/Abstract] OR minimal[Title/Abstract] OR low NIHSS[Title/Abstract] OR low National Institutes of Health Stroke Scale[Title/Abstract] OR NIHSS ≤5[Title/Abstract] OR NIHSS <6[Title/Abstract] OR NIHSS ≤3[Title/Abstract] OR NIHSS 0-5[Title/Abstract] OR NIHSS 0-3[Title/Abstract] |
| 3 | thrombolysis*[Title/Abstract] OR intravenous tissue plasminogen activator[Title/Abstract] OR rt‐PA[Title/Abstract] OR t‐PA[Title/Abstract] OR alteplase*[Title/Abstract] OR tPA[Title/Abstract] OR tenecteplase[Title/Abstract] OR TNK[Title/Abstract] OR TNKase[Title/Abstract] |
| 4 | Antiplatelet[Title/Abstract] OR Anti-platelet[Title/Abstract] OR aspirin[Title/Abstract] OR acetylsalicylic acid[Title/Abstract] OR ASA[Title/Abstract] OR Clopidogrel[Title/Abstract] OR Plavix[Title/Abstract] OR Iscover[Title/Abstract] OR thienopyridines[Title/Abstract] OR ADP receptor inhibitors[Title/Abstract] OR Ticagrelor[Title/Abstract] OR Brilique[Title/Abstract] OR Brilinta[Title/Abstract] |
| 5 | 1 AND 2 AND 3 AND 4 |

**Supplementary Table 2.** Risk of bias assessment in the included studies

| **Non-randomized Studies using Risk of Bias in Non-randomized Studies of Interventions (ROBINS-I) tool** | | | | | | | | |
| --- | --- | --- | --- | --- | --- | --- | --- | --- |
| **Study/Bias**  **domain** | **Bias due to confounding** | **Bias in selection of participants into the study** | **Bias in classification of interventions** | **Bias due to deviations from intended interventions** | **Bias due to missing data** | **Bias in measurement of outcomes** | **Bias in selection of the reported result** | **Overall** |
| Duan 2023 | Low risk of bias | Low risk of bias | Low risk of bias | Low risk of bias | Low risk of bias | Low risk of bias | Low risk of bias | **Low risk of bias** |
| Lan 2020 | Low risk of bias | Low risk of bias | Low risk of bias | Low risk of bias | Low risk of bias | Low risk of bias | Low risk of bias | **Low risk of bias** |
| Sykora 2023 | Low risk of bias | Low risk of bias | Low risk of bias | Low risk of bias | Low risk of bias | Low risk of bias | Low risk of bias | **Low risk of bias** |
| Wang 2020 | Low risk of bias | Low risk of bias | Low risk of bias | Low risk of bias | No Information | Low risk of bias | Low risk of bias | **Moderate risk of bias** |
| **Randomized clinical trials using Cochrane Collaboration’s tool for assessing the risk of bias, version 2.0 (RoB 2.0)** | | | | | | | | |
|  | **Bias in the randomization process** | **Bias in deviations**  **from intended interventions** | **Bias in missing outcome data** | **Bias in outcome measurement** | **Bias in the**  **selection of the reported results.** | **Overall** |  |  |
| Chen 2023 | Low risk of bias | Some concerns | Low risk of bias | Low risk of bias | No Information | **Some concerns** |  |  |

## Supplementary Figures


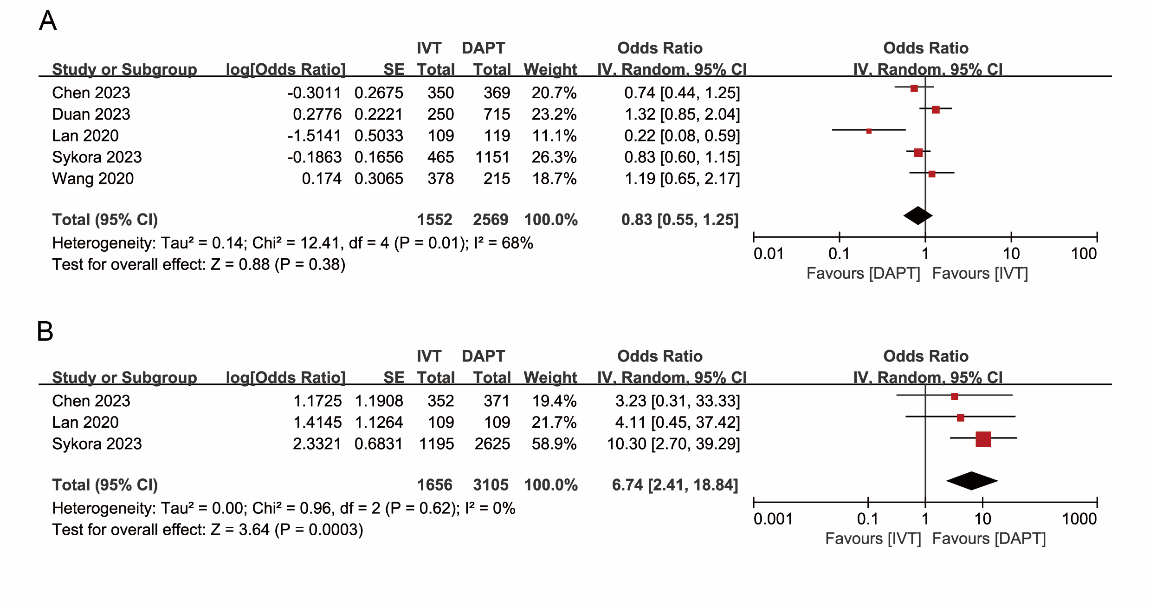


**Supplementary Figure 1** Forest plot of the adjusted odds ratios of the primary efficacy and safety outcomes in patients with acute minor ischemic stroke: (A) excellent functional outcome; (B) Symptomatic intracranial hemorrhage.


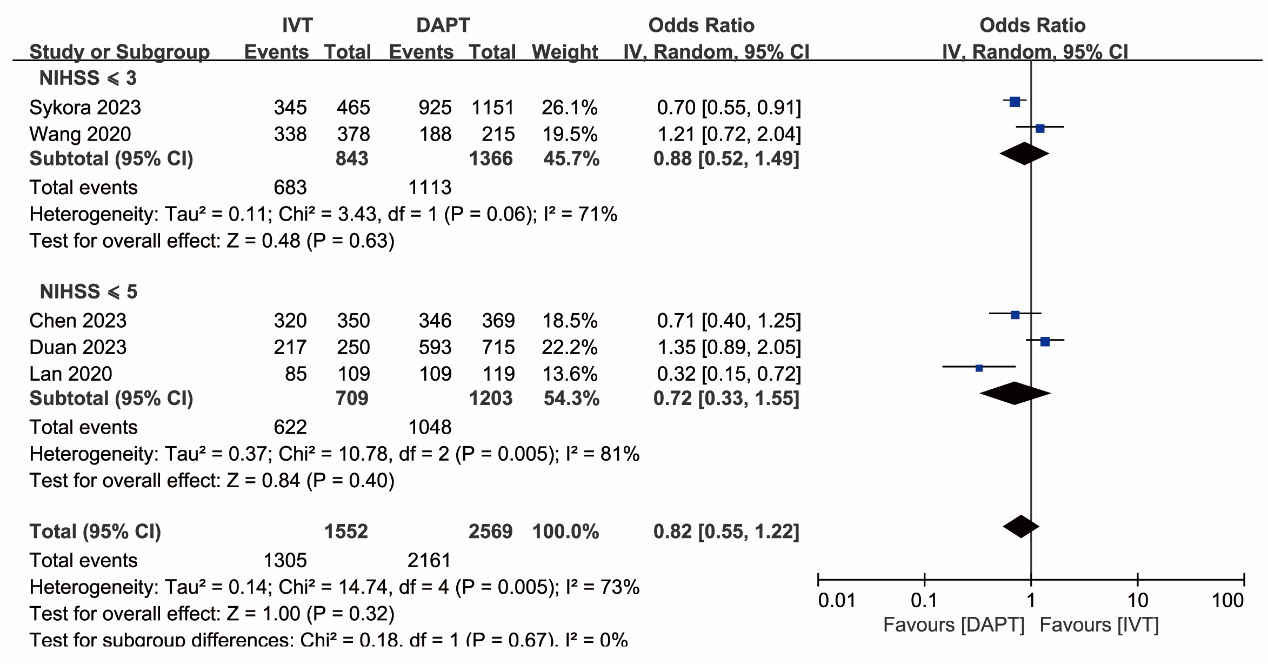


**Supplementary Figure 2** Forest plot of the subgroup analysis of NIHSS scores at admission for primary efficacy outcome (excellent functional outcome).


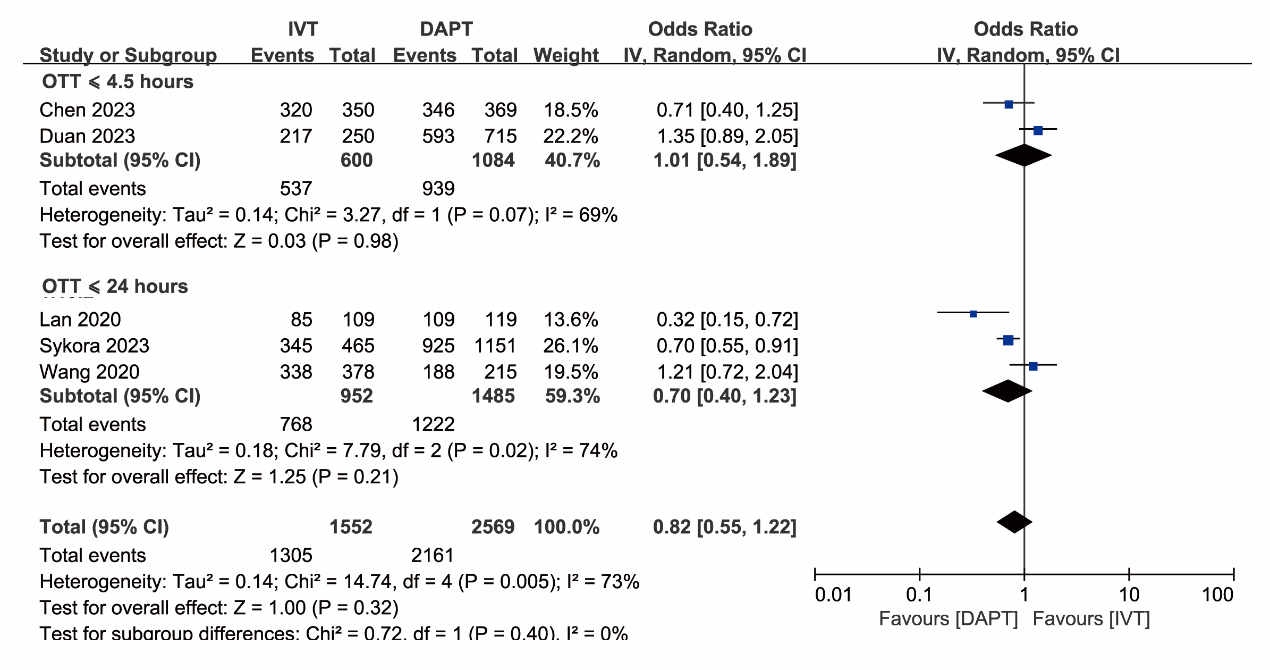


**Supplementary Figure 3** Forest plot of the subgroup analysis of onset to treatment time (OTT) into DAPT group for primary efficacy outcome (excellent functional outcome).


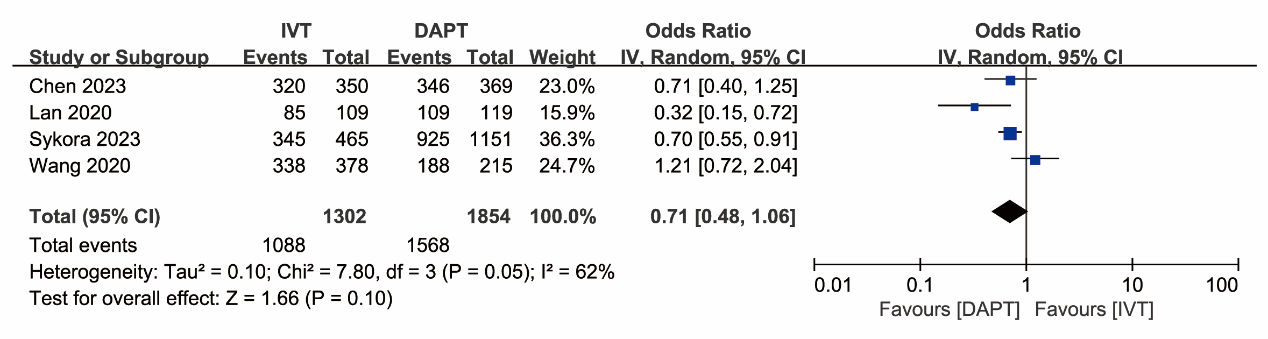


**Supplementary Figure 4** Forest plot of the sensitivity analysis by omitting the studies only included patients with LVO for primary efficacy outcome (excellent functional outcome).
